# Surgical Menopause Impairs Retinal Conductivity and Worsens Prognosis in an Acute Model of Rat Optic Neuropathy

**DOI:** 10.3390/cells11193062

**Published:** 2022-09-29

**Authors:** Edyta Olakowska, Piotr Rodak, Anna Pacwa, Joanna Machowicz, Bartosz Machna, Joanna Lewin-Kowalik, Adrian Smedowski

**Affiliations:** 1 Department of Physiology, Faculty of Medical Sciences in Katowice, Medical University of Silesia in Katowice, 40-752 Katowice, Poland; olakom@mp.pl (E.O.); piotr.rodak@sum.edu.pl (P.R.); apacwa@sum.edu.pl (A.P.); asiamach@interia.pl (J.M.); bartosz.machna@gmail.com (B.M.); jkowalik@sum.edu.pl (J.L.-K.); 2 GlaucoTech Co., 40-282 Katowice, Poland

**Keywords:** estrogens, menopause, retinal ganglion cells, apoptosis, optic neuropathy

## Abstract

Deficiency of estradiol during the menopausal period is an important risk factor for neurodegenerative diseases, including various optic neuropathies. The aim of this study was to evaluate the impact of surgical menopause on the function and survival ratio of RGCs in the rat model of ONC (optic nerve crush). We used eight-week-old female Long Evans rats, divided into two main groups depending on the time between ovariectomy procedure (OVA) and euthanasia (two weeks vs. seven weeks), and subgroups—OVA, OVA + ONC, or ONC. Retinal function was assessed with electroretinography (ERG). RGC loss ratio was evaluated using immunolabelling and counting of RGCs. Seven weeks after OVA, the menopause morphologically affected interneurons but not RGC; however, when the ONC procedure was applied, RGCs appeared to be more susceptible to damage in case of deprivation of estrogens. In our analysis, PhNR (photopic negative responses) were severely diminished in the OVA + ONC group. A deprivation of estrogens in menopause results in accelerated retinal neurodegeneration that firstly involves retinal interneurons. The lack of estrogens increases the susceptibility of RGCs to insults.

## 1. Introduction

Estrogens, in addition to their essential role in reproduction, possess various metabolic activities and are involved in a variety of aspects of human health [1]. They maintain different homeostatic and developmental processes, e.g., bone growth and turnover [2,3], glucose and lipid metabolism, brain development and cognitive functions [4]. Moreover, estrogens have stimulating and anti-inflammatory effects on the immune system, resulting in the alleviation of some autoimmune disorder risks and symptoms [5,6]. Most estrogens are produced in the ovaries; however, peripheral synthesis exists in adipose and nervous tissues and bones. Estrogens act by activating several signaling pathways via binding with two nuclear receptors—ERα (estrogen receptor α) and ERβ (estrogen receptor β)—as well as a membrane receptor, GPER (G protein-coupled estradiol receptor), and the subsequent phosphorylation and dimerization of this complex [7]. Modified complexes can bind to estrogen-responsive elements (EREs) in the promoter region of target genes and regulate their transcription [8]. ERs are expressed in various tissues, including the retina (especially in retinal ganglion cells, RGCs). RGCs are essential for the transmission of visual information processed by the photoreceptors of the retina to the brain, and RGC axons form the optic nerve [9].

Researchers have paid particular attention to age-related ocular neurodegenerative diseases because of the overlap of endocrine and neuronal dysfunction observed during aging. Hormonal decline (especially a lack of estrogens during the perimenopausal period) is an important risk factor for neurodegenerative diseases, such as glaucoma, ischemic optic neuropathy and retinopathy, age-related macular degeneration, and diabetic retinopathy [1,10,11].

Hormonal-dependent degeneration leads to the irreversible death of retinal neurons (mainly RGCs) with no possibility of regeneration. This phenomenon is especially important currently, given the increasing proportion of postmenopausal women in the worldwide population.

Estrogen supplementation in postmenopausal women significantly reduced the risk of POAG (primary open-angle glaucoma) development [12]. Moreover, the topical delivery of 17β-estradiol prevented RGC death in a glaucoma model in rats [13]. Additionally, estradiol supplementation enhanced blood flow in the retina, protected RGCs, and prevented the swelling of glial cells after ovariectomy in rats [14].

The prevalence of optic neuropathies and vascular retinal disorders increases with age (i.e., retinal vein occlusion, retinal artery occlusion, anterior ischemic optic neuropathy, diabetic retinopathy, glaucoma, anterior ischemic optic neuropathy). However, to date, there are no efficient neuroprotective treatments targeting neurodegeneration in the retina and optic nerve [15,16,17,18,19,20,21,22,23]. Since there is no efficient causative therapy for neurodegeneration, the current treatment is focused on eliminating risk factors of RGC death, but not on RGC degeneration itself [16,21,24,25,26,27]. Recent studies show involvement of Erβ receptors in the endogenous neuroprotection of axotomized RGCs via activation of the ERK-c-Fos pathway, in glaucomatous neurodegeneration via Akt/CREB/thioredoxin-1, MAPK/NF-kappaB, and inhibition of IL-18 [13,28,29]. In ischemic optic neuropathy, estrogens prevent RGCs degeneration, if applied before the trauma, with no effects of treatment post-trauma [30,31]. Estrogens can prevent the effects of oxidative insult in retinal neurons by activation of PI3K/Akt signaling and exert mitochondrial protection associated with the attenuation of the proapoptotic Bax gene [32,33]. There is evidence that topical delivery of 17β-estradiol can prevent RGCs death in a glaucoma model in rats as well as in acute axotomy model [13,29,34]. There are multiple studies showing that estrogen deficiency, associated with aging, accelerates optic nerve dysfunction [35,36].

However, estradiol has been observed as being able to protect RGCs from damage due to its antioxidant and antiapoptotic activity, and the prevention of RGC apoptosis with estrogen supplementation or estrogen receptor modulation would significantly delay vision loss in affected patients and improve their overall quality of life in the future [14].

## 2. Materials and Methods

### 2.1. Animals, Anesthesia, and Euthanasia

All experimental procedures involving animals were approved by the Local Committee for Animal Research with adherence to the European Communities Council Directive (86/609/EEC) and comparable to the guidelines published by the Institute for Laboratory Animal Research. Animals were provided by the Center for Experimental Medicine, Medical University of Silesia, in Katowice, Poland. All surgical procedures were performed under general anesthesia with an intraperitoneal injection of a mixture of ketamine (50 mg/kg, VetaKetam, Vetagro, Lublin, Poland) and xylazine (5 mg/kg, Xylapan, Vetoquinol Biowet, Pulawy, Poland). Animals were euthanized by an intraperitoneal overdose of ketamine and xylazine solution and subsequent decapitation.

### 2.2. Study Design and Study Groups

In this study, we used twenty-seven eight-week-old female Long Evans rats divided into two main groups depending on the time between the ovariectomy procedure and euthanasia. The first group consisted of 12 animals—10 were ovariectomized and 2 remained as a healthy untouched control. The animals were euthanized two weeks after ovariectomy and subsequently underwent transcardiac perfusion with 500 mL of PBS and 500 mL of ice-cold 4% PFA solution. Both eyes were collected for further retinal isolation and immunostaining. The second group consisted of 15 animals divided into 3 equal subgroups: animals that underwent an ovariectomy procedure only (OVA, *n* = 5 animals), ovariectomy and subsequent unilateral optic nerve crush in the right eye six weeks after ovariectomy (OVA + ONC, *n* = 5 animals), and animals that underwent optic nerve crush in the right eye without previous ovariectomy (ONC, *n* = 5 animals). Untreated eyes from ONC group were considered healthy. The animals were euthanized seven weeks after the ovariectomy (one week after the ONC procedure). All eyes were collected, and the retinas were isolated. In each of the three subgroups, one pair of retinas was homogenized and fractionated for further Western blot analyses, and the remaining four retinas were processed as whole mounts for immunostaining and stereology.

### 2.3. Ovariectomy Procedure

After general anesthesia, the backs of the rats were shaved, and the skin was cleaned with 70% ethanol solution (Amara, Krakow, Poland). Ovariectomy was preceded by a 2-cm long midline dorsal skin incision inferior to the rib cage. After exposing the peritoneal cavity, the adipose tissue was pulled away until the right uterine tube with the ovary was identified. The right ovary and surrounding fat were then gently retracted. After the ligation of the distal uterine horn with absorbable 4/0 Vicryl Ethicon (Johnson & Johnson, New Brunswick, NJ, USA), the right ovary was removed. Subsequently, the uterine horn was placed back into the peritoneal cavity, and the peritoneum and muscle layers were sutured with 4/0 Prolene Ethicon (Johnson & Johnson, New Brunswick, NJ, USA). The skin was sutured with 3/0 Vicryl Ethicon (Johnson & Johnson, New Brunswick, NJ, USA), and 10% povidone-iodine solution (Teva Pharmaceuticals, Warsaw, Poland) was applied to the surgical area. The left ovary was removed in the same manner described above. To avoid dehydration, all rats were subcutaneously injected with 2 mL of 0.9% sodium chloride (Polpharma, Duchnice, Poland) solution. As a painkiller, 400 mg of paracetamol (suspension 125 mg/5 mL; Polfa, Warszawa, Poland) was dissolved in 100 mL drinking water (average drug dosage, 200 mg/kg of body weight daily).

### 2.4. Optic Nerve Crush Procedure

The procedure was performed under general anesthesia. The rats were placed under the surgical microscope, and one drop of 0.5% proxymetacaine hydrochloride (Alcaine, Alcon, Fort Worth, TX, USA) was applied to the right eye for topical anesthesia. A curved pincet was gently slid between the upper and lower eyelids, and the eyeball was protruded slightly out of the orbit. Using the other hand, the conjunctiva was dissected with microscissors along the superior-temporal limbus until a 1–2 mm access to the subconjunctival space was created. The subconjunctival tunnel was enlarged with the blunt side of the scissors, and the tissues were then separated with the pincet to clearly visualize the retrobulbar space. Self-closing forceps were slid along the eyeball past the superior muscle and between large ciliary vessels, pushing away adipose tissue to expose the optic nerve. Self-closing forceps were placed around the optic nerve 0.5 mm behind the globe, and the grip was loosened and held steady for 10 s to crush the nerve. After the procedure, the tools were gently removed, and 2% chloramphenicol ointment (Detromycin 2%, Chema-Elktromet, Rzeszow, Poland) was topically applied to the eye surface and covered with a sterile pad. Animals were placed in clean cages and checked daily for any signs of infection.

### 2.5. Electroretinography

After overnight dark adaptation, the rats were generally anesthetized. One drop of 0.5% proxymetacaine hydrochloride (Alcaine, Alcon, Fort Worth, TX, USA) was applied to both eyes for topical anesthesia, and the pupils were dilated with 1% tropicamide (Polfa, Warszawa, Poland). The rats were placed on a heated platform during each recording session. ERG was recorded using a Celeris system (Diagnosys LLC, Cambridge, UK). The measurement conditions were 0.01 cds/m^2^, 0.1 cds/m^2^, 1.0 cds/m^2^, 3.0 flash, 10 flash, and 10 Hz flicker. Retinal function was evaluated using flash ERG with photopic negative response (PhNR) analysis as a function of RGCs in each animal before ovariectomy, six weeks after (before ONC) and before euthanasia (seven days after ONC). After the procedure, the animals were returned to their cages on a regular day-night cycle.

### 2.6. Immunohistochemistry

The retinas were stained using anti-β3-tubulin (Tuj1) (1:300), anti-NeuN (1:100), anti-Erα (1:500), anti-Erβ (1:500), and anti-Iba-1 (1:500) antibodies. Tissue sections were blocked for 30 min in 10% NGS/TBS solution with 0.1% Triton X-100. Appropriate dilutions of primary antibodies were applied to the specimens overnight at +4 °C. Species-matched secondary antibodies (1:500 AlexaFluor, Life Technologies, Carlsbad, CA, USA) were applied for 3 h at room temperature. Nuclei were counterstained with 4’,6-diamidino-2-phenylindole (DAPI, Sigma, Aldrich, St. Louis, MO, USA) and visualized under a Zeiss Axio Scope fluorescence microscope. A1 (Zeiss, Oberkochen, Germany).

Human retina samples from patients with POAG (*n* = 3) and healthy controls (*n* = 2) were obtained from the Human Eye Biobank for Research, St. Michael’s Hospital, University of Toronto, Canada under permission obtained from the Institutional Ethical Committee of Medical University of Silesia in Katowice, Poland. The eyeball cross-sections were deparaffinized, rehydrated and stained using anti-β3-tubulin (1:300), anti-Erα (1:500), and anti-Erβ (1:500).

### 2.7. Cell Count

Β3-Tubulin- and NeuN-positive cells were counted manually from corresponding superior-inferior quadrants of retinas using the ImageJ software (http://imagej.nih.gov/ij/, accessed on 15 September 2021). Ten photographs (five from the peripheral region and five from the central region of the retina) from each sample were taken under a 20× objective lens. The cells were counted within the ganglion cell layer of each retina, and the mean values with standard deviations were calculated.

### 2.8. Western Blot

To obtain nuclear and cytoplasmic extracts from retinas, we performed fractionation with a Nuclear Extract Kit (Active Motif, Carlsbad, CA, USA) according to the manufacturer’s instructions. The protein concentration was determined with Bradford Reagent (BioRad, Hercules, CA, USA) using the Quick Start Bovine Serum Albumin Standard Set (BioRad, Hercules, CA, USA) to obtain a standard curve. A total of 15 μg of protein concentrate was separated by 12% SDS-PAGE at 120 V and transferred onto PVDF membranes (Pall Life Sciences, New York, NY, USA) at 250 mA for 90 min. After transfer, the PVDF membranes were blocked with 3% BSA/TBS buffer for 1.5 h and incubated overnight at +4 °C with estrogen receptor β (ERβ, dilution 1:500, Cloud-Clone Corp., Houston, TX, USA) primary antibody. As a secondary antibody, we used goat anti-rabbit IgG StarBright Blue 700 (BioRad, 1:3000, Hercules, CA, USA) and incubated the membranes for 1 h in the dark at room temperature. We used Anti-tubulin hFAB Rhodamine Antibody (BioRad, 1:3000, Hercules, CA, USA) to normalize protein loading. The membrane signals were detected by multiplexed fluorescence (ChemiDoc MP, BioRad, Hercules, CA, USA). Protein bands were quantified using the Image Lab software.

### 2.9. Statistics

For statistical analysis, we used Prism 9.3.1 (GraphPad Software, Inc., La Jolla, CA, USA). Descriptive statistics are shown as the mean ± standard deviation (SD). For the pairwise comparisons, we used Welch’s *t* test, which considers unequal SDs. *p* values < 0.05 were considered significant.

## 3. Results

### 3.1. Two Weeks of Surgical Menopause Induced by Ovariectomy Led to the Loss of Retinal Interneurons but Not RGCs in the Rat Model

**Figure 1:**
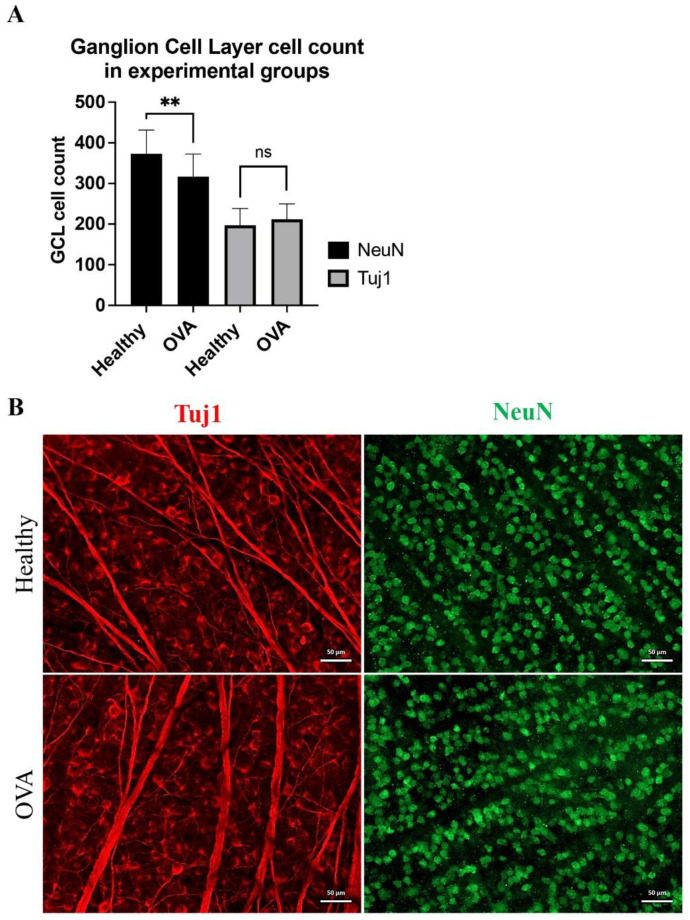
The impact of two weeks of surgical menopause on RGC and interneuron counts within the ganglion cell layer of the retina. (**A**) Statistical analysis of GCL cell count. ns: not significant, **: *p* < 0.01, Welch’s *t* test. (**B**) Immunofluorescence staining of the whole mounted retinas for Tuj1 (red) and NeuN (green). Scale bar: 50 μm.

After two weeks, the retinal cell count in the ganglion cell layer showed significant alterations. Tuj1-positive cells (presumably RGCs) showed no significant changes (197.1 ± 41.5 vs. 212 ± 37.9 for healthy and OVA, respectively, *p* > 0.05, Welch’s *t* test). The ganglion cell layer (GCL) NeuN-positive cell counts were 372.9 ± 58.3 and 316.9 ± 55.5 for the healthy and OVA groups, respectively (*p* < 0.01, Welch’s *t* test). NeuN-positive cells represent RGCs and displaced interneurons (presumably amacrine cells). The lack of significant differences in RGC counts for the Tuj1 marker suggests that NeuN alterations originate from interneuron loss after the OVA procedure (Figure 1).

### 3.2. Two Weeks of Surgical Menopause Evoked Cellular Translocation of ERβ and Increased Retinal Neuroinflammation

**Figure 2:**
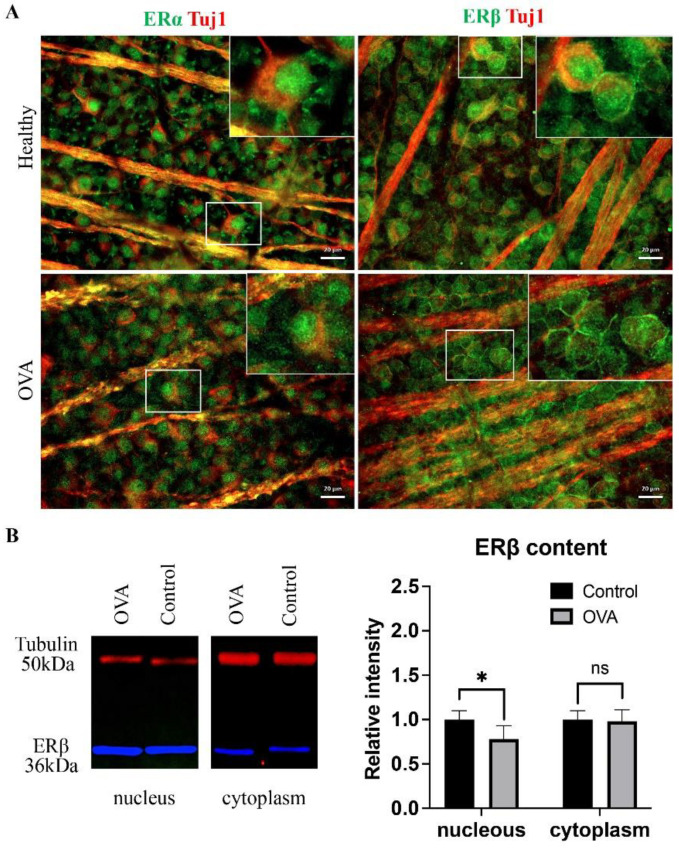

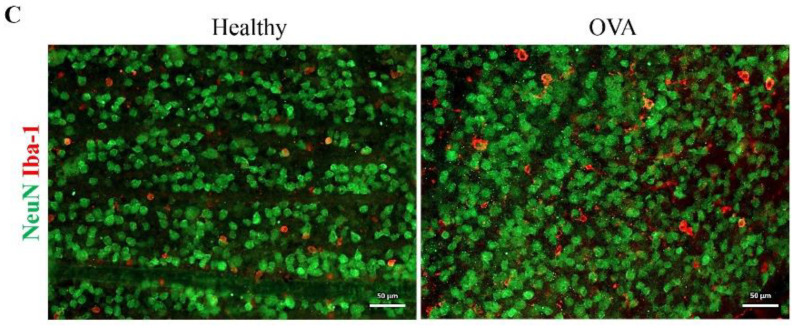
Immunofluorescence staining of whole mounted retinas. (**A**) The difference between the cellular localization of ERα and ERβ (green) colocalized with Tuj1 (red) in the healthy and OVA groups. Scale bar: 20 μm. (**B**) WB analysis of ERβ nuclear and cytoplasmic fraction in OVA and control (healthy) group, ns: not significant, *: *p* < 0.03, Welch’s *t* test. (**C**) Microglial cell infiltration positive for Iba-1 (red) double-stained with NeuN (green). Scale bar: 50 μm.

Estrogen deprivation hypersensitizes the retina to the detrimentally reduced estrogen levels by changes in estrogen receptor expression. In our experiment, the only alteration of ERα was visible as an aggregation of ERα in RGC nuclei (Figure 2A); however, ERβ showed more pronounced changes, with clear translocation of the ERβ protein from nuclei to cell surfaces (Figure 2A). The WB analysis showed increased nuclear ERβ production (*p* < 0.03, Welch’s *t* test) with a constant content of the cytoplasmic fraction (*p* > 0.05, Welch’s *t* test), which in combination with immunostaining may suggest that the translocated signal observed in the RGC originates from the cell membranes rather than the submembrane cytoplasm (Figure 2A,B). Because estrogen signaling is known to inhibit inflammation, estrogen deprivation was associated with proinflammatory cytokine release; in our study, we observed the increased infiltration of stimulated microglial cells within the retina (Figure 2C).

### 3.3. Six Weeks of Surgical Menopause Affected Retinal Interneuron Counts and Exacerbated RGC Neurodegeneration in Acute Optic Neuropathy

**Figure 3:**
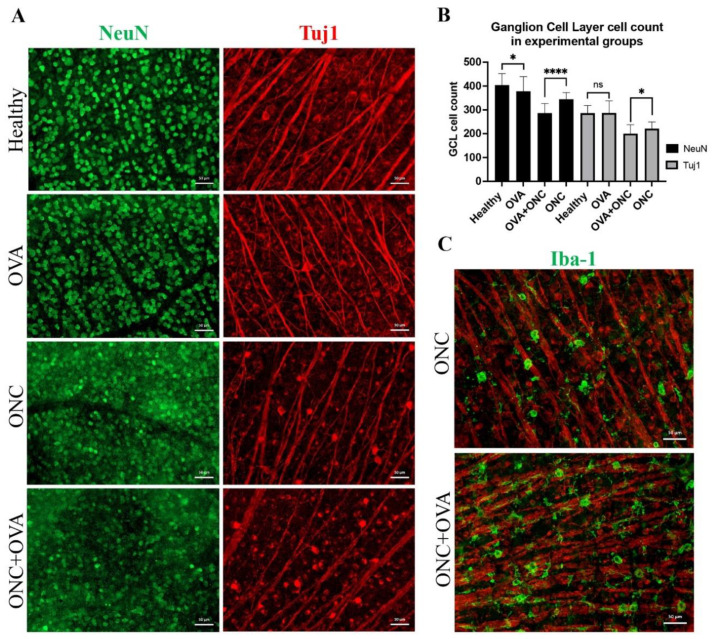
Immunofluorescence staining of whole mounted retinas. (**A**) NeuN- and Tuj1-positive cells in the GCL of whole mounted retinas. Scale bar: 50 μm. (**B**) Statistical analysis of GCL cell count, ns: not significant, *: *p* < 0.03; ****: *p* < 0.0001; Welch’s *t* test. (**C**) Iba-1 (green) cell infiltration merged with Tuj1 (red). Scale bar: 50 μm.

Extending the duration of estrogen deprivation evoked a similar pattern of retinal cell death to the two-week follow-up (Figure 3). The GCL NeuN-positive cell counts were 403.9 ± 48.5 and 378.2 ± 61 for healthy and OVA animals, respectively (*p* < 0.03, Welch’s *t* test), and the Tuj1-positive cell counts were 286.2 ± 31.7 and 286.9 ± 50.7 for healthy and OVA animals, respectively (*p* > 0.05, Welch’s *t* test).

Although surgical menopause did not affect the RGC count before any insult occurred, the importance of the neuroprotective features of estrogen signaling was clearly visible after the optic nerve crush trauma. In these settings, we observed a detrimental acceleration of RGC and interneuron degeneration, especially in the OVA + ONC group. After the acute trauma, NeuN-positive cell counts were 344.7 ± 27.7 and 286.2 ± 40 for the ONC and OVA + ONC groups, respectively (*p* < 0.0001, Welch’s *t* test). Furthermore, the Tuj1-positive cell counts were 221.4 ± 27.7 and 200.3 ± 37.5 for the ONC and OVA + ONC groups, respectively (*p* < 0.03, Welch’s *t* test). Estrogen deprivation, together with neuropathic trauma (ONC) induced visible RGC axonopathy that could be observed as a dotted axonal structure in Tuj1 staining (Figure 3). Estrogen deprivation increased macrophage infiltration after optic nerve crush (Figure 3).

### 3.4. The Electrical Function of Different Populations of Retinal Neurons Was Compromised by Both Ovariectomy and Optic Nerve Crush

**Figure 4:**
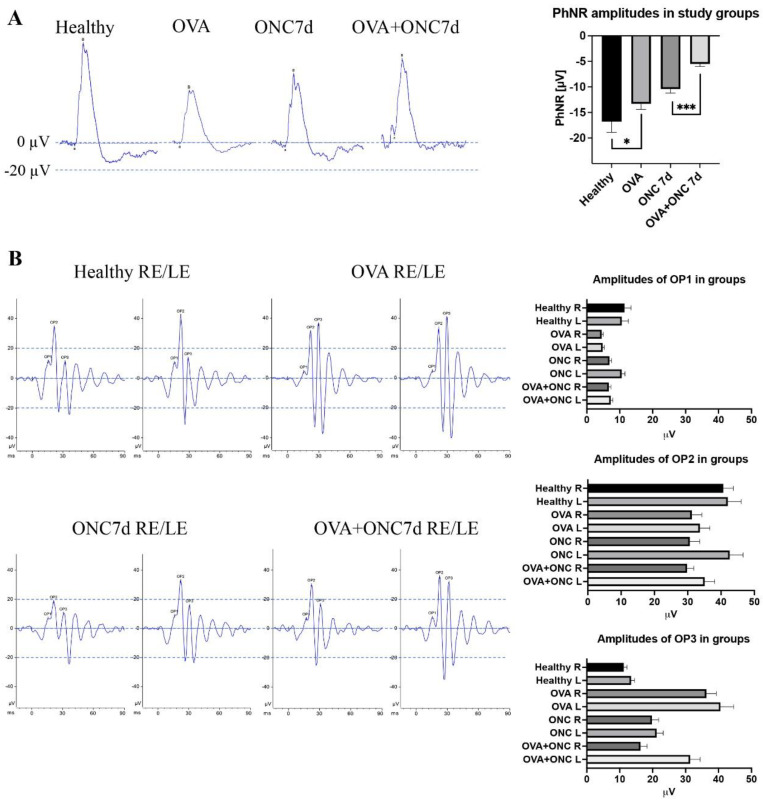
Functional measurements in animal groups using ERG. (**A**) Photopic negative responses (PhNR) in experimental groups, including statistical analysis of PhNR amplitudes, *: *p* < 0.03, ***: *p* < 0.001; Welch’s *t* test. (**B**) Oscillatory potentials (OP) in experimental groups including descriptive statistics for OP1, OP2 and OP3. RE: right eye, LE: left eye.

In the six-week menopause group, the PhNR responses, which indicate the activity of retinal ganglion cells, were −16.8 ± 2.1 and −13.3 ± 1.1 μV for the healthy and OVA groups, respectively, (*p* < 0.03, Welch’s *t* test) and −10.4 ± 0.8 and −5.5 ± 0.5 μV for the ONC and OVA + ONC groups, respectively (*p* < 0.001, Welch’s *t* test) (Figure 4A). The PhNR recordings proved that although morphologically unaffected, surgical menopause deeply impaired the function and endogenous neuroprotective capacities of RGCs.

An early oscillatory potential (OP1-3) analysis allowed us to draw conclusions about the functional condition of retinal interneurons (mostly amacrine cells—OP1-2) and the general vascular status of the retina (retinal glia, retinal vessels, and blood flow—OP3). The latencies of OPs showed no significant differences between groups. The amplitudes of OP1 were 11.42 ± 2/10.61 ± 1.93 (right eye/left eye), 4.5 ± 0.49/4.8 ± 0.5, 6.9 ± 0.6/10.6 ± 1.1, and 6.7 ± 0.6/7.3 ± 0.58 μV for the healthy, OVA, ONC and OVA + ONC groups, respectively. The amplitudes of OP2 were 40.68 ± 3.1/42.02 ± 4.04; 31.21 ± 3.15/33.66 ± 3.18; 30.6 ± 2.91/42.6 ± 4.12; and 29.8 ± 2.1/35.1 ± 3.18 μV for the healthy, OVA, ONC and OVA + ONC groups, respectively. The amplitudes of OP3 were 11.22 ± 1.1/13.46 ± 1.13; 36.31 ± 2.93/40.6 ± 4.08; 19.8 ± 2.1/21.22 ± 2.21; 16.3 ± 1.97/31.4 ± 3.27 μV for the healthy, OVA, ONC and OVA + ONC groups, respectively (Figure 4B).

### 3.5. Human Glaucomatous Retinas Revealed Alterations in Estrogen Receptor Expression and Cellular Localization

**Figure 5:**
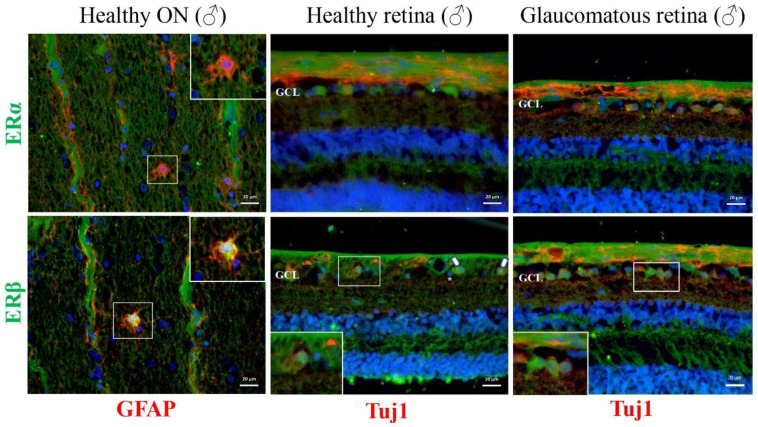
Immunofluorescence staining of longitudinal sections of healthy human optic nerve and healthy and glaucomatous retinal cross sections. In the optic nerve sections, ERα and ERβ are labeled in green, colocalizing with GFAP in red. In the retinal cross sections, ERα and ERβ are labeled in green, colocalizing with Tuj1 in red. Scale bar: 20 μm. Arrow: indicates nuclear expression of ERβ within RGCs, asterisk: nuclear expression of ERβ within GCL interneurons, GCL: ganglion cell layer.

In the healthy male optic nerve and retina, the major ER receptor was ERβ, similar to the optic nerves and retinas of female rats (Figure 2A and Figure 5). ERβ expression was visible in the cell bodies of optic nerve astrocytes and in retinal ganglion cells and ganglion cell layer interneurons in the retina. The localization of ERβ in healthy RGCs was clearly nuclear. In contrast, ERβ translocated into the membrane compartment in the glaucomatous group (Figure 5).

## 4. Discussion

Similar to brain neurons, retinal neurons are affected by aging and become more susceptible to inflammation, impaired recovery, and neurodegenerative diseases, such as glaucoma [10]. The goal of our study was to evaluate the impact of surgical menopause on visual functions in a rat model of acute optic neuropathy. In our study, we attempted to characterize morphological changes in the retina of ovariectomized rats by assessing the RGC and interneuron counts. Additionally, we conducted a complex electrophysiological evaluation of RGC and retinal interneuron function. To this end, we introduced two surgical models, ovariectomy and optic nerve crush. This experimental model of optic nerve crush allows us to precisely study RGC death and can be standardized in animals [37,38,39,40,41].

In our experiments, we used a well-established rat model of surgical menopause induced by the bilateral removal of ovaries, which leads to a rapid decline in circulating estrogens [42]. First, we showed that two weeks of surgical menopause caused significant a loss of retinal interneurons (presumably amacrine cells) but not RGCs. In another approach, we showed that six weeks of menopause impaired the function of both interneurons and RGCs; however, it did not affect the RGC count. When an additional trauma (optic nerve crush) was introduced, surgical menopause sensitized RGCs to damage, resulting in accelerated apoptosis and diminishing PhNR responses. This degeneration of retinal neurons developed because of a decrease in estradiol, which is neuroprotective.

Nakazawa et al. observed that the density of surviving RGCs after optic nerve injury in ovariectomized rats was significantly lower than that in rats without ovariectomy in the same model of nerve damage. Moreover, treatment with estradiol reduced the loss of RGCs by inhibiting apoptosis. This finding indicates that ovariectomy alone does not affect the survival of RGCs in rats if the estradiol levels are equated [29]. Allen et al. investigated the impact of eight weeks of surgical menopause on the possible exacerbation of visual dysfunction in a rat model of RGC injury induced by optic nerve crush. They revealed that ovariectomy worsened visual acuity (a decrease in spatial frequency and contrast sensitivity). It also induced a thinning of the retinal nerve fiber layer in all ovariectomized rats. The authors observed that surgical menopause can affect the function of RGCs after injury [43]. Similarly, Feola et al. revealed that surgical menopause worsened visual function in rats with increased ocular hypertension. They also observed a thinning of retinal fiber layer and a loss of total retinal thickness in rats with unilateral ocular hypertension [44]. In another study, the same authors showed that surgical menopause in rats affected some physiological factors (outflow facility and ocular compliance) associated with glaucoma pathogenesis. They suggested that such estrogen deprivation can contribute to a higher risk of glaucoma in postmenopausal women [45]. In the current study, we identified both estradiol receptors, ERα and ERβ, in RGCs. In healthy retina and optic nerve fibers, nuclear ERβ was the main type of estrogen receptor. However, after ovariectomy, we observed a translocation of ERβ from the nuclear to the membrane compartment. This translocation could be linked to the hypersensitivity of cells to critically reduced estrogen levels and has also been described by other authors [46]. Changes in ERα expression in our study consisted only of receptor aggregation in RGC nuclei. Some authors state that ERβ has a dominant role in neurodegenerative diseases and can be a potential target for the treatment of retinal injury [35]. Cascio et al. observed that ERα is mainly present in amacrine cells and RGCs, whereas ERβ is predominant in the inner synaptic layer of the retina [47]. Hence, the neuroprotective activity of 17-β estradiol was shown to be mediated by ERβ, but not ERα, in a model of oxidative stress induced in the retinal pigment epithelium. This neuroprotection occurred via the preservation of mitochondrial function, reduction of reactive oxygen species synthesis, and induction of antioxidant genes [48]. Several different isoforms of ERβ with possible roles in different cellular processes have been described [49,50]. In our study, the isoform detected in WBs of retinal homogenates appeared at approximately 35 kDa, which has been reported previously; however, this isoform has a role in the regulation of other estrogen receptors rather than in ligand binding [51,52].

In addition to its obvious participation in the apoptosis of retinal neurons, we also observed that estrogen deficiency increased the infiltration of stimulated microglial cells (macrophages) within the retina. This phenomenon may be associated with a lack of anti-inflammatory activity of estrogens [53,54]. Microglial cells are the first line of defense against damage in the brain and retina and play a key role in the progression of neuroinflammation and neurodegeneration in glaucoma [55,56]. Many studies have suggested that estradiol can diminish proinflammatory cytokine synthesis and protect RGCs against neuroinflammatory damage and that its loss may trigger microglial activation. This process is the first step of neural injury observed in glaucoma and can directly induce RGC loss [57,58]. In humans, the application of estrogen receptor modulators, i.e., raloxifene, alleviates neuroinflammation after traumatic brain injury [59].

The neuroprotective role of estrogens has been investigated in various neurodegenerative diseases, such as glaucoma. Hulsman et al. observed that early menopause (natural menopause or menopause after irradiation therapy or bilateral ovariectomy) in women is associated with a higher risk of open-angle glaucoma [60]. In addition, the risk for the onset of glaucoma was found to be significantly increased in women undergoing bilateral ovariectomy before the age of 43, and even hormonal replacement therapy with estradiol did not reduce this risk [61]. Li et al. showed that decreased levels of estradiol and increased levels of IL-8 in postmenopausal women were associated with a higher risk of primary angle closure glaucoma and faster progression of the disease [62].

In our study, electroretinography provides a new understanding of the impact of estrogen deficiency on retinal function. Ovariectomy itself notably decreased the whole photopic ERG amplitudes, suggesting impairment in intraretinal signal conduction. In the complex ERG analysis, we found that the most affected group of retinal neurons in the case of selective OVA were interneurons, for which we observed both morphological and functional decline. In the case of RGC, the functional decline preceded the morphological loss, which became visibly exacerbated after optic neuropathy trauma. This finding may suggest that retinal interneurons may be the population of retinal neurons that are especially sensitive to estrogen loss, which, to our knowledge, has not been reported before. In the oscillatory potential analysis, we found evidence of photoreceptor and interneuron declines after the ovariectomy procedure, as observed in OP1-2 amplitude decreases [63,64]. However, the increase in OP3 amplitudes after ovariectomy may suggest both the stimulation of retinal glia and an impact on the retinal blood flow related to the differences in the rheological features of blood and vascular resistances in postmenopausal animals [11,65,66,67].

Many authors have reported that estrogen treatment may prevent RGC degeneration and promote survival, enhance blood flow in the retina, preserve visual acuity, and decrease the risk of developing glaucoma [12,13,14]. Moreover, estrogen treatment has been mentioned as a future possible therapeutic strategy for the treatment of this neurodegenerative disease [68].

In our study, we evaluated the influence of surgical menopause on the visual function of RGCs after optic nerve crush injury. The results show that estrogen loss caused by ovariectomy induced RGC degeneration and a loss of interneurons as well as neuroinflammatory reactions in the retina. These data are consistent with scientific evidence showing that estrogen deprivation increases the risk of retina and optic nerve degeneration.

Changes in estradiol levels during women’s lives can affect the function of RGCs and worsen visual function and optic nerve viability. Diminished exposure to the hormone can correlate with an increased risk of retinal neurodegeneration and glaucoma development. While a progressive loss of RGCs, optic nerve injury and the exacerbation of retinal conductivity are the main causes of glaucoma, the precise mechanism of RGCs death in this disease remains unclear; thus, investigating all possible pathways involved in disease pathogenesis is important.

## Data Availability

The datasets used and/or analyzed during the current study are available from the corresponding author on reasonable request.
