# Correction: Olakowska et al. Surgical Menopause Impairs Retinal Conductivity and Worsens Prognosis in an Acute Model of Rat Optic Neuropathy. *Cells* 2022, *11*, 3062

**DOI:** 10.3390/cells14151175

**Published:** 2025-07-30

**Authors:** Edyta Olakowska, Piotr Rodak, Anna Pacwa, Joanna Machowicz, Bartosz Machna, Joanna Lewin-Kowalik, Adrian Smedowski

**Affiliations:** 1 Department of Physiology, Faculty of Medical Sciences in Katowice, Medical University of Silesia in Katowice, 40-752 Katowice, Poland; olakom@mp.pl (E.O.); piotr.rodak@sum.edu.pl (P.R.); apacwa@sum.edu.pl (A.P.); asiamach@interia.pl (J.M.); bartosz.machna@gmail.com (B.M.); jkowalik@sum.edu.pl (J.L.-K.); 2 GlaucoTech Co., 40-282 Katowice, Poland

In the original publication [1], the funding information was incorrect. The funding information “Polish National Science Center Preludium, grant no. 2021/41/N/NZ4/01271” was removed. The updated funding statement is provided below. The authors state that the scientific conclusions are unaffected. This correction was approved by the Academic Editor. The original publication has also been updated.
